# NMDA Receptor Antagonist Ketamine Impairs Feature Integration in Visual Perception

**DOI:** 10.1371/journal.pone.0079326

**Published:** 2013-11-01

**Authors:** Julia D. I. Meuwese, Anouk M. van Loon, H. Steven Scholte, Philipp B. Lirk, Nienke C. C. Vulink, Markus W. Hollmann, Victor A. F. Lamme

**Affiliations:** 1 Department of Psychology, University of Amsterdam, Amsterdam, The Netherlands; 2 Cognitive Science Center Amsterdam, University of Amsterdam, Amsterdam, The Netherlands; 3 Department of Anesthesiology, Academic Medical Center, University of Amsterdam, Amsterdam, The Netherlands; 4 Department of Psychiatry, Academic Medical Center, University of Amsterdam, Amsterdam, The Netherlands; Ecole Polytechnique Federale de Lausanne, Switzerland

## Abstract

Recurrent interactions between neurons in the visual cortex are crucial for the integration of image elements into coherent objects, such as in figure-ground segregation of textured images. Blocking N-methyl-D-aspartate (NMDA) receptors in monkeys can abolish neural signals related to figure-ground segregation and feature integration. However, it is unknown whether this also affects perceptual integration itself. Therefore, we tested whether ketamine, a non-competitive NMDA receptor antagonist, reduces feature integration in humans. We administered a subanesthetic dose of ketamine to healthy subjects who performed a texture discrimination task in a placebo-controlled double blind within-subject design. We found that ketamine significantly impaired performance on the texture discrimination task compared to the placebo condition, while performance on a control fixation task was much less impaired. This effect is not merely due to task difficulty or a difference in sedation levels. We are the first to show a behavioral effect on feature integration by manipulating the NMDA receptor in humans.

## Introduction

Making sense of a visual scene requires integration of features such as luminance, orientation, motion and contrast. As visual input enters the brain, it is propagated in a hierarchical, feedforward manner by visual areas with increasing receptive field sizes, extracting increasingly complex information [1]. Subsequently, higher-order areas modulate activity of neurons in early visual areas via feedback connections [2]. This contextual modulation seems to be crucial for feature integration, as it enables neurons in early areas to change their signaling based on information beyond their receptive fields [3].

A fundamental visual process requiring feature integration is figure-ground segregation. In order to differentiate a figure from its background, feedback connections between higher and lower order visual areas are thought to be essential [4–7], together with long-range lateral connections within a visual area [8–10]. The relative contributions of feedback and lateral connections are presently not fully disentangled [11–13], therefore we refer to the combination of lateral and feedback interactions as ‘recurrent processing’ [14]. As early as in V1, recurrent connections enable neurons to respond more strongly to a figure than to a background texture, even when both present identical local features to the receptive field and while the figure is much larger than that receptive field [4,6].

Figure ground segregation can be manipulated in several ways. Recurrent interactions can be effectively disrupted by presenting a mask shortly after the visual stimulus, rendering the stimulus invisible [15,16]. Furthermore, applying Transcranial Magnetic Stimulation (TMS) to V1 at approximately 100 ms after stimulus presentation has been shown to impair figure-ground segmentation [17]. A similar TMS-induced disruption has been shown for orientation and color perception [18].

Interestingly, feature integration can also be selectively abolished by anesthesia. Although there are a wide variety of anesthetics, in general they cause depression of neural activity by reducing excitatory glutamatergic neurotransmission and potentiating inhibitory GABAergic neurotransmission [19,20]. Disrupting this balance between excitation and inhibition seems to selectively affect recurrent interactions [21,22]. Monkeys anesthetized with isoflurane (binding to GABA, NMDA and glycine receptors) showed fully suppressed contextual modulation related to figure-ground segregation, whereas feedforward activity remained unaffected [10]. Li et al. [23] found that contour integration responses in V1 disappeared under anesthesia using pentobarbital (acting at the GABAa receptor). 

Although many anesthetics target inhibitory GABA receptors, some agents predominantly act at excitatory receptors, like the NMDA-receptor [24]. Modeling studies have proposed that these NMDA receptors mediate recurrent processing [25,26]. This idea is supported by the fact that the NMDA receptor has some distinctive properties. NMDA channel opening is voltage-dependent; it only opens when the magnesium blockade is removed by sufficient prior depolarization caused by AMPA receptors (thought to carry the feedforward signal) [27,28]. This unique property might explain the modulatory function of NMDA receptors, as it ensures amplification of the firing rate of neurons that are driven by feedforward connections [29]. Although most excitatory glutamatergic synapses contain both AMPA and NMDA receptors, NMDA ratios seem to be higher at synapses targeted by recurrent connections, in supragranular layers of the visual cortex [14,30–32]. Optical imaging showed that NMDA receptors boost the horizontal spread of excitation in supragranular layers of primary visual cortex of rats, suggesting that NMDA plays a role in integrating neural responses [33,34]. In addition, NMDA currents take much longer to activate compared to the very fast AMPA currents, but their decay is much slower. Because of this extended time course, their total contribution to recurrent excitation is thought to be at least twice as high as that of AMPA currents [35,36]. Supporting this idea, a monkey study showed that blocking the NMDA receptor with 2-amino-5-phosphonovalerate and Ifenprodil in primary visual cortex reduced figure-ground modulation, whereas blocking AMPA receptors did not [14].

Together, these findings suggest that the balance between excitatory and inhibitory neurotransmission plays an important role in neural feature integration processes leading to figure-ground segregation. More specifically, the NMDA receptor seems to be involved in mediating recurrent processing. However, it is unclear whether manipulating these modulatory signals in the brain affects feature integration at the perceptual level. NMDA receptor blocking in the monkey experiments was fairly local and restricted to V1, and the figures were designed to be well above the threshold for perception [14], so that a behavioral effect on figure-ground perception was neither expected nor found. 

Here we use ketamine, a non-competitive NMDA antagonist [37,38], and a more sensitive behavioral paradigm, to examine its effect on feature integration in humans. Ketamine primarily targets NMDA receptors, and is often used to study the role of NMDA receptors in human cognition (see for a review, 39), although it may have minor additional effects on various other signaling pathways (see ‘Discussion’ section for details). We administered a subanesthetic dose of ketamine to healthy subjects who performed a texture discrimination task of increasing difficulty [40]. This task requires figure-ground segregation in order to detect whether line elements are horizontally or vertically aligned. Performance was compared to a placebo condition using a double blind within-subject design, and to performance in a fixation task to control for the general effects of subanesthesia. The aim of our study is twofold: to see whether the NMDA-blocker induced effects in monkeys can be scaled up to humans, to a systemic rather than local application, and to see whether this indeed results in selective impairments in figure-ground segregation at the perceptual level.

## Methods

### Ethics Statement

The experiment was approved by the Medical Ethical Review Committee of the Academic Medical Center of Amsterdam. We obtained written informed consent from each participant before experimentation. Our dataset is freely available upon request.

### Participants

Twenty subjects (11 males, 9 females) participated in the experiment for financial compensation. Participants were screened by a psychiatrist for psychiatric disorders, drug addiction and physical conditions that may cause complications upon ketamine administration. Participants had normal or corrected-to-normal vision, two were left-handed. Participants were required to refrain from recreational drug usage for 30 days prior to participation and to have had no prior experience with ketamine. Four subjects were excluded because of incorrect task order administration and technical failure. All analyses are based on the remaining sixteen participants (10 males, 6 females, *M* = 23.21 years of age, SD = 1.17 years).

### General Procedure

The experiment was a within-subject, double blind, placebo-controlled design. Subjects completed two sessions, two weeks apart: one during which ketamine was administered and one where a placebo was administered. The order of the drug conditions (ketamine or placebo) was counterbalanced across subjects. One week prior to the start of the experiment a practice session took place to familiarize subjects with the task. In both sessions, subjects performed a texture discrimination task in which they had to discriminate the orientation (horizontal or vertical) of a peripheral target texture that was masked at a decreasing stimulus onset asynchrony (SOA). The quadrant in which the target texture appeared (upper left or right) was counterbalanced across conditions and sessions. To monitor subjective sedation levels, subjects filled in visual analogue scales (VAS) indicating their mood [41] at four time points (see ‘VAS’ section below for details).

 The experiment was part of a larger set of studies on ketamine-induced impairments (including resting-state BOLD measurements, working memory, motion induced blindness and perceptual learning tasks), of which the results are not reported here. The content of these separate studies was equal for each session and condition.

### Task design

**Figure 1:**
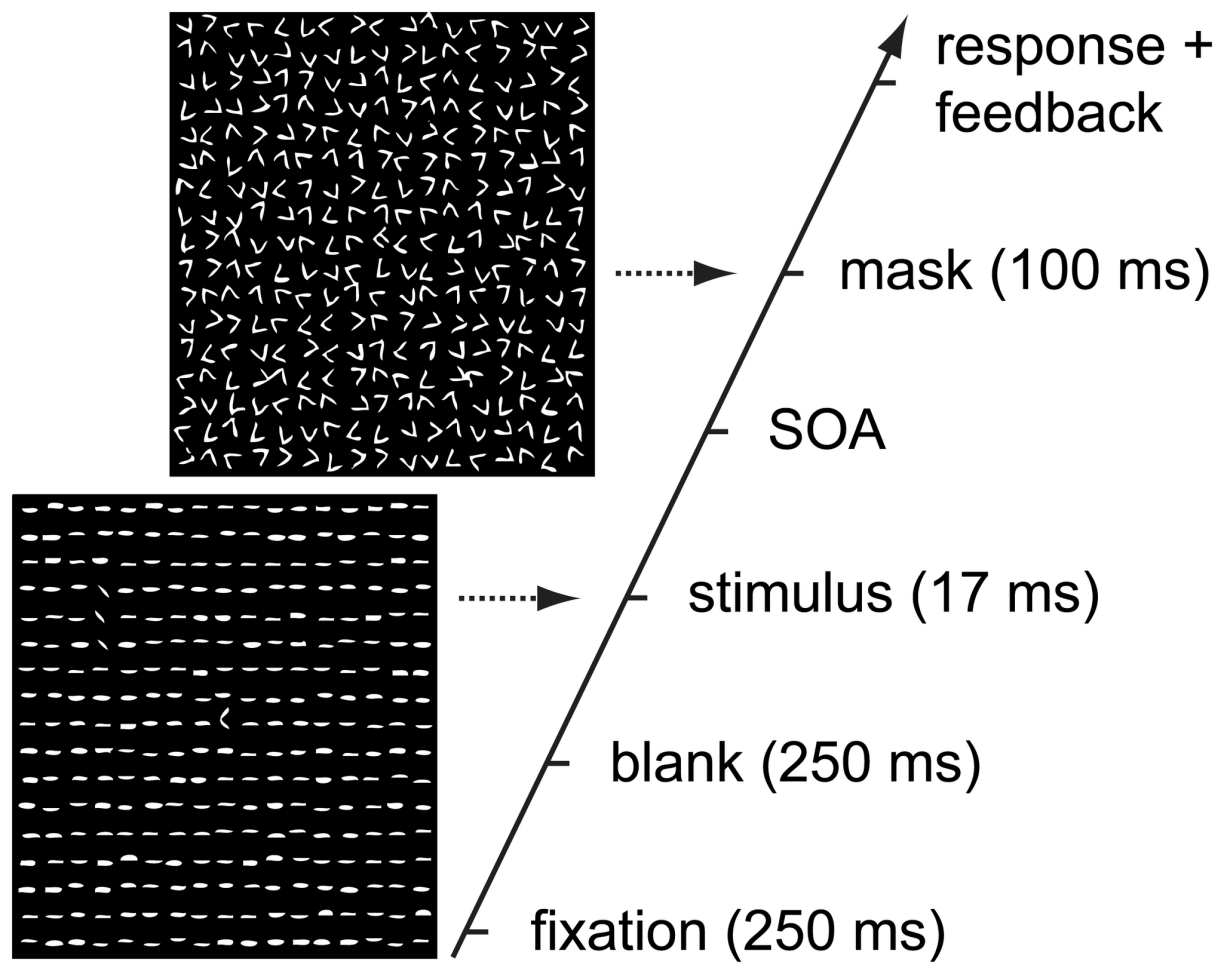
Schematic illustration of a single trial. On each trial, subjects performed a central letter identification task (a randomly rotated T or L) to ensure fixation (‘fixation task’) and identified the orientation of a peripheral target texture (horizontal or vertically aligned line segments) (‘texture discrimination task’). SOA between stimulus and mask decreased from 467 to 66 ms. Immediate auditory feedback was provided for the fixation task. Figure was adapted with permission from Schwartz et al. [43].

On each trial, subjects performed a dual task, first designed by Karni and Sagi [40]. Subjects had to identify a central letter (a randomly rotated T or L) (‘fixation task’), whilst discriminating the orientation of a masked peripheral target texture (horizontally or vertically aligned line segments) (‘texture discrimination task’) (see Figure 1). The central task ensured good fixation, whereas the texture discrimination task measured feature integration. As SOA’s between stimulus and mask decrease, the task becomes increasingly difficult.

Stimuli consisted of a square (13 x 13°) containing 17 x 17 jittered horizontal line elements (0.43 x 0.07°, spaced 0.76° apart). The target consisted of three diagonally oriented line elements, aligned in either a horizontal row or a vertical column, appearing at random locations (always in either the upper left or right quadrant, counterbalanced across subjects and drug condition), 2.5-5° from the central letter. After each stimulus a mask was presented (at variable SOA intervals), which impeded target discrimination. The mask consisted of randomly oriented V-shapes (in a similar but jittered 17 x 17 configuration as the stimulus) and a central pattern of a superimposed T and L (see Figure 1). A chin rest was used to restrict head movements. A Viewsonic LCD screen was used with a refresh rate of 60 Hz (40 x 26° of visual angle). Stimuli were presented using Matlab (Mathworks, MA, USA) together with the Cogent toolbox (developed by the Cogent 2000 team at the Functional Imaging Laboratory (FIL) and Institute of Cognitive Neuroscience (ICN), University College London, UK).

We used two versions of the task: a standard version, and a practice version (used one week before the start of the experiment). The practice version of the task consisted of blocks containing 10 trials each, with an ‘easy’ SOA of 600 ms. After every block a screen was presented showing the percentage correct on both tasks (fixation and texture discrimination). The practice session terminated after subjects performed 90% correct or higher on both tasks. The standard version consisted of 14 blocks containing 26 trials each (preceded by one practice block containing 10 trials with an SOA of 600 ms, which was disregarded from our analysis). The time interval between stimulus and mask slowly decreased between blocks, SOA’s were set at 467, 367, 267 and 167 ms (one block each) and 133, 117, 100, 83 and 66 ms (two blocks each). These SOA’s were chosen based on a pilot study showing a steady decrease of performance with these decreasing SOA’s. Immediate auditory feedback was provided for the fixation task, to ensure high task performance (and thus fixation). No feedback was given for the texture discrimination task. For both tasks chance performance is at 50% correct.

For each drug condition (ketamine or placebo), the target texture was always presented in the same quadrant (upper left or right [42], counterbalanced across subjects). As improvements in task performance do not transfer across quadrants [40,43,44], this enabled us to assess texture discrimination in both conditions whilst limiting within-subject transfer effects of task performance. In the practice session the target texture was presented in yet another quadrant (lower left) for all subjects.

Reaction times were recorded on all trials, from mask onset to the first (fixation task) and second (texture discrimination task) button press. Mask onset was chosen instead of the more common stimulus onset, to prevent longer SOA’s between stimulus and mask to automatically cause longer reaction times.

### Drug administration

Venous access was established using a 20G intravenous catheter (Vasofix, B Braun, Melsungen, Germany). Subsequently, drugs were administered intravenously by an anesthesiologist unblinded to group allocation for safety reasons. The anesthesiologist was not involved in task administration. Experimenters and subjects were blind to the drug condition.

In the ketamine condition, a subanesthetic dose of S-ketamine (Eurocept BV, Ankeveen, The Netherlands) was administered intravenously. First a slow bolus of 0.15 mg/kg was administered, followed by continuous infusion of 0.1 mg/kg/h using an infusion pump (Perfusor fm, B Braun, Melsungen, Germany) to keep plasma ketamine levels constant throughout the experiment. In the placebo condition, saline (NaCl 0.9% (B Braun, Melsungen, Germany)) was administered via the same procedure. The time between initial drug administration and the beginning of testing was two hours, as in between subjects took part in a separate study (not reported here, see ‘General Procedure’ for details).

### VAS

In order to monitor subjective effects of drug administration during each session, subjects filled in visual analogue scales (VAS) [41] at different time points (before drug administration (baseline), after first (bolus) administration, before starting the task, and after testing). Each scale consisted of a 100 mm line, connecting two opposite states of mind (e.g. ‘alert’ and ‘drowsy’). Subjects were to mark a point on the scale that best indicated their state, which was then quantified as the distance to that mark measured from the left end of the scale (in mm) (e.g. if the mark is 60mm away from ‘alert’ (and 40mm from ‘drowsy’), the subject feels more drowsy than alert and has a drowsiness score of 60). The subjective state of sedation during the experiment was calculated as the mean score on a subset of these scales (alert-drowsy, excited-calm, clear headed-muzzy, energetic-lethargic, quick-slow) [45]. VAS is a frequently used measure of mood states, that has been tested for validity and reliability [46–48].

### Exit interview

In an exit interview subjects answered questions about possible side effects of drug administration, whether they had been able to carry out all tasks as required and whether they knew during which session they had received ketamine.

### Data analysis

SOA curves were constructed for performance and reaction time by averaging over subjects per drug condition per SOA. For SOA’s that were used for two consecutive blocks (when the SOA was set at 133, 117, 100, 83 and 66 ms), values were averaged across blocks, resulting in one value per SOA. Performance and reaction time curves were compared using a repeated measures ANOVA for drug condition (ketamine, placebo) x SOA (467, 367, 267, 167, 133, 117, 100, 83 and 66 ms). Drug order (whether subjects received ketamine during the first or the second session) was included as a between-subjects factor. We included subjective sedation scores (ketamine minus placebo sedation score, rated at the start of the task) as a covariate in our repeated measures ANOVA’s, to control for (individual differences in) sedative effects of ketamine.

Trials with a reaction time deviating more than two SD from the average on the fixation task or the texture discrimination task were excluded from analysis. This ensures that both reaction time and performance effects are not driven by any outlier (extremely slow or quick) reaction time trials, where subjects may not have been performing the task properly.

Sedative effects of ketamine and placebo were analyzed comparing each sedation score with the baseline sedation score, and comparing each score between drug conditions, all using paired t-tests. Statistical analysis was performed using SPSS 17.0 (IBM, Armonk, USA).

## Results

### Performance

**Figure 2:**
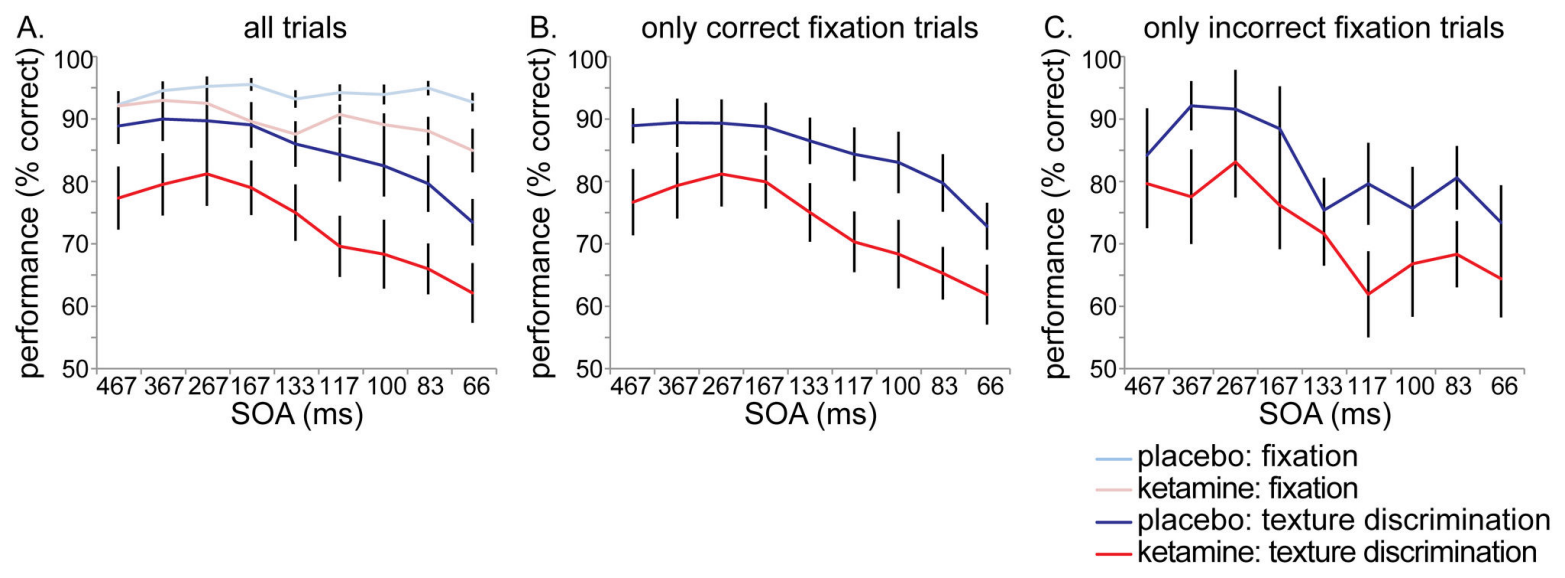
Task performance. Performance on all trials, for the fixation task and texture discrimination task (A). Performance on the texture discrimination task including only correct fixation trials (B) and including only incorrect fixation trials (C). Ketamine administration caused decreased performance on both tasks, but texture discrimination impairments were independent of fixation task performance (impairments were equal for correct and incorrect fixation trials).

As hypothesized, performance on the texture discrimination task was impaired in the ketamine condition compared to the placebo condition (F_(1,13)_ = 14.667, *p* = .002). However, performance on the fixation task was also affected in the ketamine condition (F_(1,13)_ = 12.068, *p* = .004) (see Figure 2A).

Since we are specifically interested in the texture discrimination task (as a measure of feature integration), we also analyzed performance including only trials where subjects performed correctly on the fixation task (see Figure 2B). This enabled us to compare performance on the texture discrimination task between conditions whilst performance on the fixation task was equal, and it ensures central fixation during the texture discrimination task. When only correct fixation trials were included, ketamine still significantly reduced performance on the texture discrimination task (F_(1,13)_ = 13.678, *p* = .003).

Decreased performance under ketamine administration was not merely an effect of task difficulty. There was a general effect of SOA on performance on the texture discrimination task, indicating that the task became increasingly difficult with decreasing SOA’s between stimulus and mask (F_(8,6)_ = 4.723, *p* = .037). However, the ketamine-induced performance impairment was not enhanced with increasing task difficulty, as there was no interaction of drug condition and SOA on the texture discrimination task (F_(8,6)_ = .557, *p* = .78). In fact, performance in the ketamine condition was steadily impaired throughout the task, from the start (at an ‘easy’ SOA of 467 ms) onwards. For the fixation task there was no main effect of SOA (F_(8,6)_ = 1.463, *p* = .33) and no interaction of SOA and drug condition either (F_(8,6)_ = .684, *p* = .70). Thus for both conditions, performance on the fixation task did not decrease with increasing task difficulty, indicating that the ketamine-induced impairments are not an unspecific result of task difficulty.

It could be argued that impaired performance on the texture discrimination task is caused by dual task interference: perhaps performing well at the primary (fixation) task happens at the expense of performance on the secondary (texture discrimination) task. Therefore, we also analyzed performance including only *incorrect* fixation trials (see Figure 2C). Also on these trials, ketamine significantly reduced performance on the texture discrimination task compared to placebo administration (F_(1,13)_ = 11.714, *p* = .005). In fact, in both conditions texture discrimination performance is equally affected for both correct and incorrect fixation trials (ketamine condition: F_(1,13)_ = .329, *p* = .58; placebo condition: F_(1,13)_ = 2.377, *p* = .15). As texture discrimination performance is impaired independent of fixation task performance, it seems to be a specific impairment of feature integration instead of mere dual task interference.

**Figure 3:**
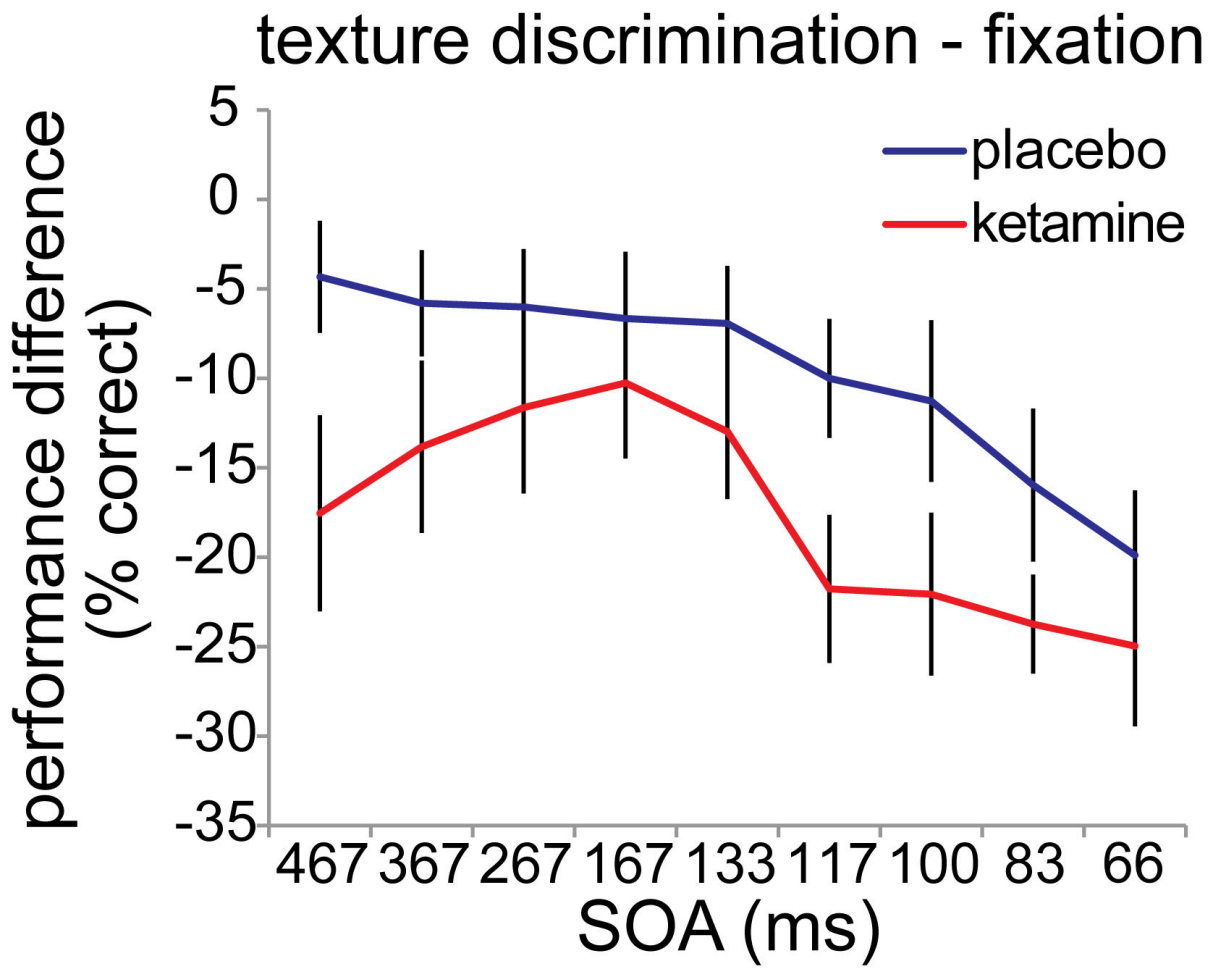
Performance difference between ketamine and placebo condition. In the ketamine condition performance on the texture discrimination task is significantly more affected than performance on the fixation task, compared to the placebo condition (F_(1,13)_ = 9.026, *p* = .01).

Furthermore, we tested whether the ketamine-induced impairment of texture discrimination is larger than the fixation task impairment. Therefore, we ran an ANOVA on the performance difference between the texture discrimination task and the fixation task, for the ketamine versus the placebo condition (see Figure 3). A main effect of drug condition was observed (F_(1,13)_ = 9.026, *p* = .01). This indicates that in the ketamine condition performance on the texture discrimination task is indeed significantly more affected than performance on the fixation task, compared to the placebo condition. No effect of SOA was found (F_(8,6)_ = 2.670, *p* = .12) and no interaction of drug condition and SOA (F_(8,6)_ = .434, *p* = .86).

### Side effects

**Figure 4:**
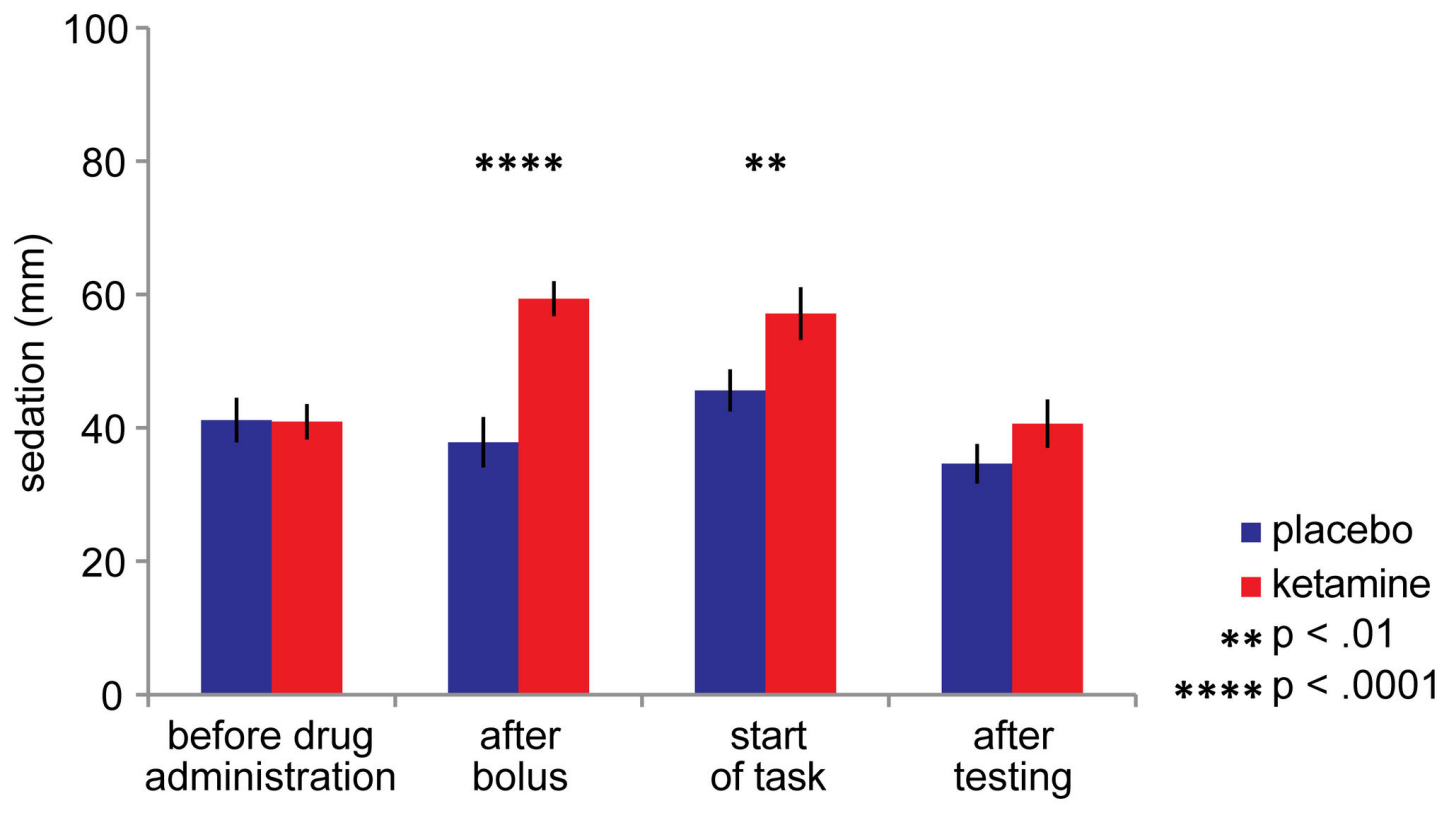
Subjective state of sedation during ketamine and placebo administration. Sedation level was based on a subset of the visual analogue scales (VAS) [41,45], filled in at different time points of the experiment. It is quantified as the distance (in mm) from a mark placed by the subject on each scale (a 100 mm line connecting two opposite states of mind), measured from the left end of the scale (see ‘Methods’ for details). Sedation levels differed between drug conditions after bolus administration (t_(1,15)_ = -5.563, *p* = .00005) and at the start of the task (t_(1,15)_ = -2.957, *p* = .0098). Sedation levels did not differ before drug administration (t_(1,15)_ = .083, *p* = .94), but after testing subjects in the ketamine condition were still feeling slightly more sedated compared to the placebo condition (t_(1,15)_ = -2.087, *p* = .054, at trend level). Within-condition, sedation levels after bolus administration and at the start of the task differed from before drug administration for the ketamine (all t_(1,15)_> 5.6, all *p* < .0005) but not for the placebo condition (within-condition p-values not depicted in this figure).

**Figure 5:**
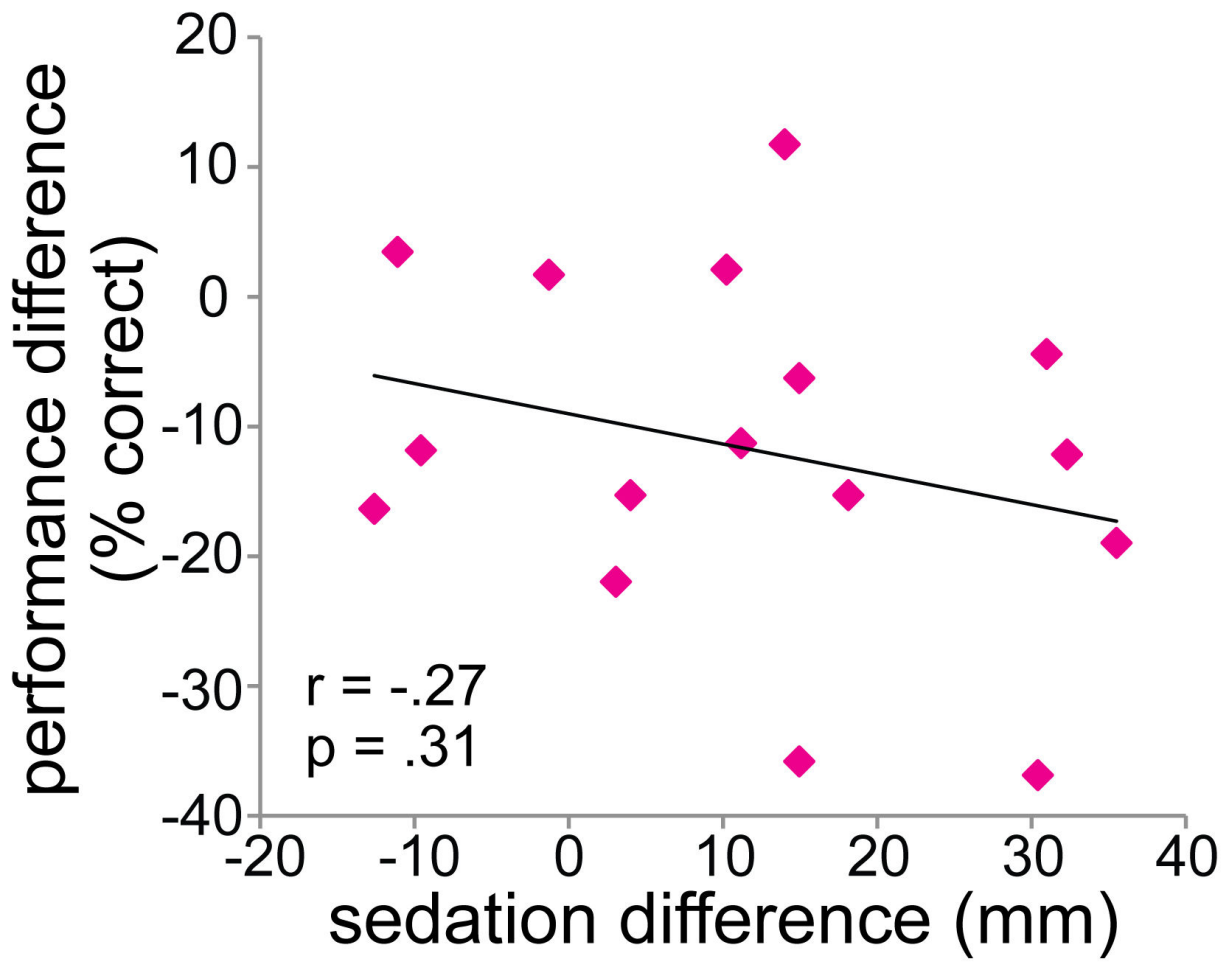
Pearson’s correlation between the sedative effect of ketamine and ketamine-induced performance impairment. The sedative effect of ketamine is measured as the sedation score, rated at the start of the task (ketamine minus placebo condition. It is quantified as the distance (in mm) from a mark placed by the subject on each scale (a 100 mm line connecting two opposite states of mind), measured from the left end of the scale (see ‘Methods’ for details). The ketamine-induced performance impairment is calculated as the average performance on the texture discrimination task (ketamine minus placebo condition). No significant correlation was observed.

Subjects felt significantly more sedated in the ketamine condition compared to the placebo condition (at the start of the task: t_(1,15)_ = -2.957, *p* = .0098, see figure 4). However, the ketamine-induced performance impairment was not directly caused by sedation, as there is no correlation between sedation level at the start of the task (ketamine minus placebo) and texture discrimination performance impairment (ketamine minus placebo) (Pearson’s r = -.27, *p* = .31, see Figure 5).

Even though the sedative effect of ketamine was not significantly correlated with ketamine-induced performance impairments, we included sedation as a covariate in our ANOVA’s (see ‘Performance’ section) to control for (individual differences in) sedative effects of ketamine. In all of these ANOVA’s, sedation level did not interact with drug condition (all F_(1,14)_ < 1.4, all p > .26).

**Figure 6:**
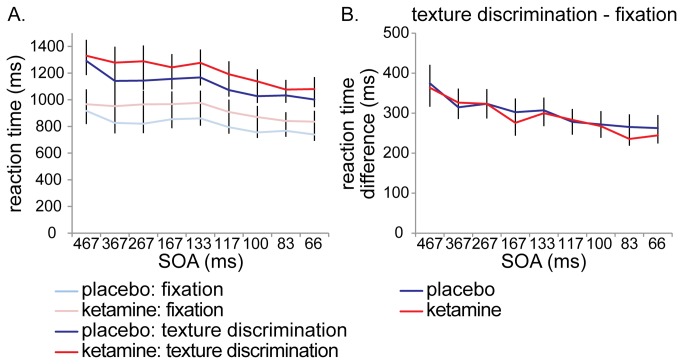
Reaction times. Reaction times on the texture discrimination task and on the fixation task were not affected during ketamine administration, indicating that ketamine-induced performance impairments were not caused by sedation (A). Ketamine administration did not slow down responses to the texture discrimination task (compared to the fixation task) any further than the placebo condition (B).

Reaction times on the texture discrimination task were not affected during ketamine administration, further supporting the notion that performance impairments were not due to the sedative effect of ketamine (F_(1,13)_ = .767, *p* = .40). The same applies for the fixation task (F_(1,13)_ = 1.153, *p* = .30). There was an overall effect of SOA, subjects became faster as SOA’s decreased (texture discrimination task: F_(8,6)_ = 20.260, *p* = .001; fixation task: F_(8,6)_ = 4.897, *p* = .034), but no interactions of drug condition and SOA were observed (texture discrimination task: F_(8,6)_ = 1.744, *p* = .26; fixation task: F_(8,6)_ = .426, *p* = .87) (see Figure 6A). When reaction time differences between the texture discrimination task and the fixation task were compared, it turned out that subjects in the ketamine condition were not slowed down by the texture discrimination task any further than in the placebo condition (F_(1,13)_ = .227, *p* = .64) (see Figure 6B). This was the case throughout the task, as there were no interactions of drug condition with SOA (F_(8,6)_ = .455, *p* = .85), and no main effect of SOA either (F_(8,6)_ = 2.314, *p* = .16). The fact that reaction times and reaction time differences between tasks are not affected by ketamine, further supports the idea that ketamine administration does not simply affect texture discrimination performance due to task difficulty or enhanced dual task interference.

In the exit interview, the following side effects of ketamine were most reported: reduced visual acuity, dizziness and confusion (two-thirds of the subjects), restlessness and nausea (one-third of the subjects). All subjects were able to carry out the tasks as required, although one-third of the subjects under ketamine administration found it difficult to concentrate. Although the experiment was double-blind (subjects were not told which drug condition they were in), in the exit interview it became apparent that all subjects were aware that they had been administered ketamine during the ketamine condition. Apparently, ketamine administration has such an effect on subjects that in practice, it is impossible for subjects to remain unaware of their condition. This is one of the reasons why drug order is counterbalanced across subjects. 

## Discussion

In this study, we tested whether ketamine reduced perceptual integration in humans using a within-subjects double blind placebo-controlled design. We found that a subanesthetic dose of ketamine significantly impaired performance on a texture discrimination task compared to a placebo condition. This seems to be not merely an effect of task difficulty, as ketamine impaired overall performance, and this impairment was not enhanced any further by increasing task difficulty (as SOA’s decreased). Although subjects were significantly more sedated in the ketamine condition, this did not directly correlate with performance impairments, and the found effects are controlled for differences in sedation levels.

As ketamine is a NMDA receptor blocker, we think that the found ketamine-induced disrupted feature integration further supports the evidence that recurrent processing is mediated via NMDA receptors. These results are in line with modeling studies [25,26] and electrophysiological recordings in monkeys showing that NMDA receptor blockers other than ketamine (2-amino-5-phosphonovalerate and Ifenprodil) reduced figure-ground modulation [14]. A similar impairment of ketamine-induced feature integration has been shown in rats by Kurylo and Gazes [34]. They find reduced perceptual grouping of spatially isolated stimulus elements (rows of dots forming lines), and claim that this is caused by disrupting NMDA-mediated lateral interactions [33]. We are the first to show a behavioral effect on feature integration by manipulating the NMDA receptor in humans.

Remarkably, in a previous backward-masking study [49] no effect was observed after administration of NMDA receptor antagonist dextromethorphan. These conflicting results could be due to a combination of dose, task differences between the experiments and pharmacological profile. Although NMDA receptors are the primary target for ketamine, ketamine has a complex pharmacological profile and it exerts some additional effects on various other signaling pathways, such as dopamine, opioid, mono-aminergic, cholinergic, nicotinic, muscarinic, serotonergic, GABAergic and AMPA receptors [50–52]. Those interactions may also contribute to the observed effects, and the differential effects observed for ketamine and dextrometorphan (that has multiple mechanisms of action as well, such as sigma-1 receptor antagonism [53]). Namely, as explained in the introduction, blocking the NMDA receptor is not the only way in which feature integration can be manipulated. There are many other neurotransmitters involved in the balance between excitation and inhibition underlying recurrent interactions, which can be targeted using different drugs [19]. For instance, the GABAa receptor agonist lorazepam impaired figure detection in humans, affecting late ERP activity while leaving early activity relatively intact, disturbing recurrent excitatory connections balanced by GABAergic interneurons [49]. The muscarinic antagonist scopolamine targeting acetylcholine receptors has been shown to reduce attentional feedback in V1, by recording single cell activity in monkeys [54]. On the other hand, scopolamine in a lower dose did not affect figure detection in humans [49], so the extent of these effects remains unclear.

In a low dose however, such as used in the current study (0.1 mg/kg/h), ketamine seems to be a relatively selective and potent antagonist of the NMDA receptor [55,56], and neuromodulatory transmitters seem to have little effect. Even at a much higher dose (10 mg/kg) no effect on dopamine, noradrenaline and serotonine levels was found in PFC or striatum of rats *in vivo* [50,57]. Acetylcholine (ACh) levels were increased in PFC and hippocampus of rats for a ketamine dose ranging from 10 to 100 mg/kg [58–60], however no increase was found for a dose of 2 mg/kg [60]. Considering the (relatively) high ketamine concentrations needed to increase the level of neuromodulatory transmitters, this seems unlikely to have contributed to our results. Furthermore, at a low dose (0.5 mg/kg/h), ketamine does not seem to have cholinergic receptor affinity in humans [61,62].

Interestingly, ketamine was recently discovered to be a potent and rapid antidepressant [63], due to downstream consequences of NMDA antagonism. Ketamine disinhibits GABAergic input to glutamate-containing neurons, enhancing presynaptic glutamate release [64]. Because of the NMDA receptor blockade, glutamatergic transmission through AMPA then increases relative to NMDA [65–67]. This increased stimulation of AMPA receptors eventually results in a rapid increase of synaptogenesis [68–70]. The antidepressant effect of a single, subanesthetic dose of ketamine was notable within two hours [71], which was the exact onset between initial ketamine administration and the beginning of testing in the current study. Although in these studies a slightly higher dose of ketamine is applied (0.5 mg/kg), it is unclear whether the increased activation of AMPA receptors relative to NMDA receptors may have affected our results.

Dose-dependent effects of ketamine on sensory perception were reported previously as assessed via a questionnaire [72]. Using the same dose as our study (0.1 mg/kg), one-third of the subjects reported visual disturbances such as blurred vision and visual field narrowing (‘tunnel vision’). In our exit interview, two-thirds of the subjects reported to experience reduced visual acuity. These reports of visual disturbances might be explained by our findings of diminished feature integration during ketamine administration. Furthermore, recurrent interactions are not only thought to be involved in feature integration, but in attention and awareness as well [73,74]. Perhaps ketamine-induced reduced feature integration results in reduced awareness as well. We hypothesize that this reduction of awareness starts with peripheral vision. Alternatively, it could be the other way around: the visual disturbances could be unrelated to feature integration but nonetheless underlie decreased performance on our texture discrimination task.

It could be argued that our finding of impaired figure-ground segregation in the ketamine condition is due to a general effect of task difficulty. However, if impaired performance would be related to task difficulty alone, one would expect increasingly worse performance under ketamine administration compared to the placebo condition as the task becomes more difficult (at shorter SOA’s), indicated by an interaction of drug condition x SOA. There is no such interaction, performance in the ketamine condition is worse than the placebo condition throughout the whole task on a steady level, from the start (at an ‘easy’ SOA of 467 ms) onwards. The fact that no reaction time differences are found between conditions further supports the idea that ketamine-induced performance impairments are not caused by task difficulty. Furthermore, a study varying load from low to high in two different working memory tasks did not find any effects of load on performance in the ketamine condition, suggesting that ketamine-induced effects are not simply a result of increasing task difficulty [75].

As our experimental design consists of a dual task, the possibility cannot be ruled out that our effects are not task-specific, but merely caused by ketamine-induced enhanced dual task interference. Even though the texture discrimination performance impairments seem independent of fixation task performance, to fully exclude the possibility of a non task-specific effect we suggest that in follow-up research a separate control task of similar difficulty should be included. For instance, it could be the case that ketamine differentially affects attention, limiting attentional resources to the secondary (texture discrimination) task. The evidence for attentional effects of ketamine is however limited and not consistent. Ketamine has been known to impair sustained attention at doses higher than used in the current study (yet still subanesthetic) [76–81]. However, others report no deficits [82–84], claiming that sustained attention is unaffected when the task does not load on working memory. Selective attention measured by tasks such as the Stroop task seems not to be affected by ketamine, even at higher doses [84–88]. A study that covaried attentional effects found that episodic memory effects of ketamine were still significant [77], suggesting that at least episodic memory deficits are not simply caused by attentional deficits [89]. Little is known about the effect of ketamine specifically on dual task interference. One study using a divided attention task (requiring differential responses to an auditory and a visual stimulus) found no effect of a low dose of ketamine (0.18 mg/kg/h, which is higher than ours), although performance was partly affected by a high dose (0.3 mg/kg/h) [81].

In sum, we found impaired perceptual integration under administration of ketamine, compared to a placebo condition. Possibly, this is related to reduced recurrent input, carried by feedback connections from higher to lower visual areas and/or lateral connections within a visual area. We hypothesize that these effects are caused by blockade of NMDA receptors, as that is the principal site of action of ketamine, and these receptors are thought to be involved in recurrent interactions. However, ketamine is known to act at other binding sites as well, which may have contributed to these results. 
